# Tribenzoatobismuth(III): a new ­polymorph

**DOI:** 10.1107/S1600536810035543

**Published:** 2010-09-11

**Authors:** Nikolay A. Tumanov, Evgenia V. Timakova, Elena V. Boldyreva

**Affiliations:** a REC–008, Novosibirsk State University, Pirogova 2, Novosibirsk 630090, Russian Federation; b Institute of Solid State Chemistry and Mechanochemistry SB RAS, Kutateladze 18, Novosibirsk 630128, Russian Federation

## Abstract

A new polymorph (β) was obtained for an active pharmaceutical ingredient, bis­muth tribenzoate, [Bi(C_6_H_5_CO_2_)_3_]. The new β-polymorph is 1.05 times denser than the previously known polymorph [Rae *et al.* (1998 ▶). *Acta Cryst.* B**54**, 438–442]. In the β-polymorph, the Bi atom is linked with three benzoate anions, each of them acting as a bidentate ligand, and these assemblies with *C*_3_ point symmetry can be considered as ‘mol­ecules’. The structure of the β-polymorph has no polymeric chains, in contrast to the previously known polymorph. The ‘mol­ecules’ in the β-polymorph are stacked along [001], so that the phenyl rings of the neighbouring mol­ecules are parallel to each other. Based on the pronounced difference in the crystal structures, one can suppose that two polymorphs should differ in the dissolution kinetics and bioavailability.

## Related literature

The synthesis of the complex is described by Timakova *et al.* (2010 ▶). For background to bismuth complexes, see: Mehring (2007 ▶); Kislichenko (1999 ▶); Goddard *et al.* (2003 ▶); Alcock (1972 ▶). For the previously known polymorph, see: Rae *et al.* (1998 ▶). For related structures, see: Hanawalt *et al.* (1938 ▶); Rosmann *et al.* (1995 ▶).
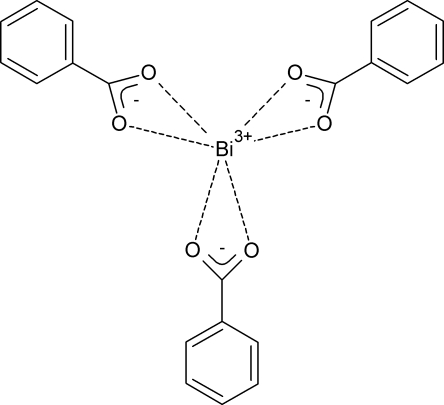


## Experimental

### 

#### Crystal data

[Bi(C_7_H_5_O_2_)_3_]
*M* *_r_* = 572.31
Trigonal, 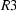

*a* = 19.5608 (19) Å
*c* = 4.0967 (5) Å
*V* = 1357.5 (2) Å^3^
*Z* = 3
Mo *K*α radiation
μ = 9.77 mm^−1^
*T* = 295 K
0.07 × 0.01 × 0.01 mm

#### Data collection

Oxford Diffraction Gemini R Ultra diffractometer
Absorption correction: multi-scan (*CrysAlis PRO*; Oxford Diffraction, 2010 ▶) *T*_min_ = 0.954, *T*_max_ = 1.000
3909 measured reflections
1355 independent reflections
1076 reflections with *I* > 2σ(*I*)
*R*_int_ = 0.087

#### Refinement

*R*[*F* ^2^ > 2σ(*F* ^2^)] = 0.042
*wR*(*F* ^2^) = 0.049
*S* = 0.76
1355 reflections
84 parameters
H-atom parameters constrained
Δρ_max_ = 1.52 e Å^−3^
Δρ_min_ = −0.68 e Å^−3^
Absolute structure: Flack (1983 ▶), 622 Friedel pairs
Flack parameter: −0.034 (12)

### 

Data collection: *CrysAlis PRO* (Oxford Diffraction, 2010 ▶); cell refinement: *CrysAlis PRO*; data reduction: *CrysAlis PRO*; program(s) used to solve structure: *SHELXS97* (Sheldrick, 2008 ▶); program(s) used to refine structure: *SHELXL97* (Sheldrick, 2008 ▶); molecular graphics: *Mercury* (Macrae *et al.*, 2006 ▶); software used to prepare material for publication: *publCIF* (Westrip, 2010 ▶) and *enCIFer* (Allen *et al.*, 2004 ▶).

## Supplementary Material

Crystal structure: contains datablocks global, I. DOI: 10.1107/S1600536810035543/rk2224sup1.cif

Structure factors: contains datablocks I. DOI: 10.1107/S1600536810035543/rk2224Isup2.hkl

Additional supplementary materials:  crystallographic information; 3D view; checkCIF report

## Figures and Tables

**Fig. 1.**
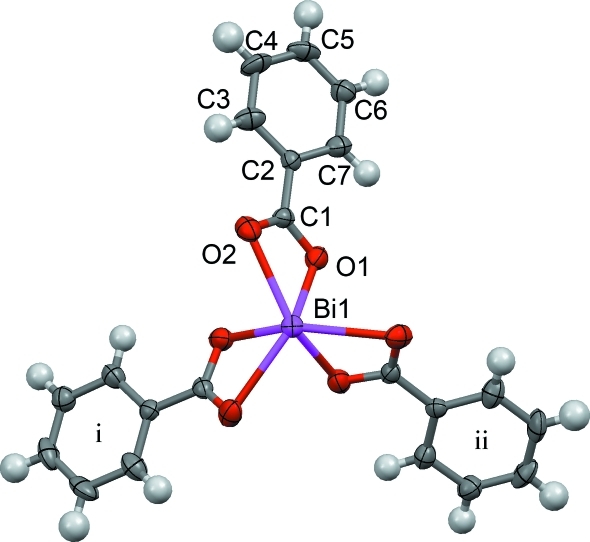
An ellipsoid plot of the title compound, showing the atom–numbering scheme and 50% probability displacement ellipsoids. Hydrogen atoms were shown as s small spheres of arbitrary radii and were not labeled for clarity. Symmetry codes: (i) -1-x+y, 1-x, z; (ii) 1-y, 2+x-y, z.

**Fig. 2.**
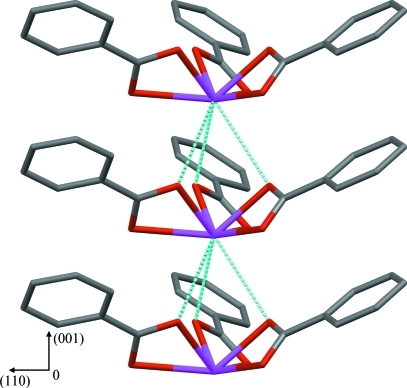
The stacking of bismuth tribenzoate `molecules' along [001] in the title compound. Intermolecular Bi1···O1 contacts shown as blue dashed lines. Hydrogen atoms were omitted for clarity.

**Fig. 3.**
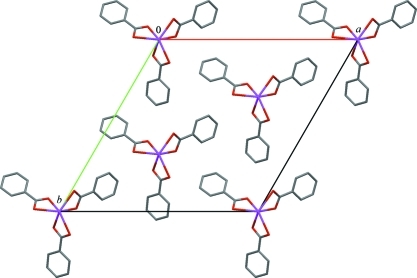
The crystal packing of the title compound viewed along the c axis. Hydrogen atoms were omitted for clarity.

## supplementary crystallographic information

### Comment

Bismuth tribenzoate, as other bismuth salts, is an active pharmaceutical ingredient, which has anti–infective and analgesic effects on lymphatic tissue and mucous tunic (Kislichenko, 1999), (Goddard *et al.*, 2003). Besides, it can be used as precursor for fine–dispersed powders of the metallic bismuth and its oxides (Mehring, 2007).

While developing a new method of synthesis of the title salt (Timakova *et al.*, 2010), we have discovered, that the powder diffraction pattern of the compound obtained in our experiments I did not match the one calculated based on the single–crystal diffraction data for the known monoclinic polymorph of bismuth tribenzoate, which was prepared by anionic exchange of bismuth triacetate with benzoic acid (Rae *et al.*, 1998). At the same time, the powder diffraction pattern of I agreed well with the powder diffraction pattern reported in a much earlier publication (Hanawalt *et al.*, 1938). We have managed to select a small crystal from the batch of the synthesized compound I of quality suitable for a single–crystal diffraction study. The powder diffraction pattern calculated based on the results of the structure solution using this single–crystal agreed well with the diffraction pattern of the whole polycrystalline batch, thus proving, that the crystal was representative for the whole sample. We report herein the crystal structure of the β-polymorph of bismuth tribenzoate.

The new β-polymorph I is 1.05 times denser than the previously known polymorph II with the space group *P*2_1_/*m* (Rae *et al.*, 1998): the density of I at room temperature is 2.098 g/cm^3^, and that of II at 173 K is 1.99 g/cm^3^. The structure of I is isomorphous to the structure of antimony tribenzoate (Rosmann *et al.*, 1995). In I each bismuth atom is linked to three benzoate anions, which act as bidentate ligands (Bi1—O1 distance is 2.254 (5)Å) and Bi1—O2 distance is 2.513 (5)Å). These assemblies with *C*_3_ point symmetry can be considered as `molecules' (Fig. 1). The `molecules' in I are stacked along [0 0 1], so that the phenyl rings of the neighbouring `molecules' are parallel to each other (Fig. 2). The carboxylate group in a ligand is rotated at 13.3 (3)° relative to phenyl ring. The neighbouring `molecules' can be supposed to interact noticeably with each other, as far as one can judge from the intermolecular Bi1···O1^i^ [symmetry code: (i) *x*, *y*, *z*-1] distances (3.110 (6)Å), which are shorter, than the sum of van der Waals radii of Bi and O (3.67Å; Alcock, 1972). Still, the structure of I has no polymeric chains, in contrast to the II. Phenyl ring of each benzoic anion is placed between two benzoic cations of neighbouring `molecules' (Fig. 3). In the structure of II each bismuth atom is also linked to three benzoate ligands, however, O atoms act as bridges between the neighbouring Bi atoms, thus forming infinite polymeric chains along *a* axis. No individual molecules can be selected in II. Based on the pronounced difference in the crystal structures, one can suppose that two polymorphs should differ in the dissolution kinetics and bioavailability.

### Experimental

The title salt was precipitated from bismuth perchloric acid solution (prepared from 217 ml of distilled water, 483 ml of concentrated perchloric acid and 850 g of bismuth oxide, then diluted to 1:10 with water) by benzoic acid with the mole ratio of benzoate–ions to bismuth being equal to 3. The reaction was carried out at 343 K during 1 h. The obtained white powder of bismuth tribenzoate was filtered and washed once with water and dried at room temperature.

Analysis found: Bi 36.6 (7), C 43.9 (4), H 2.65 (4); calculated Bi 36.5, C 44.07, H 2.64.

The typical particle size in the powder sample was in the range 0.002–0.01 mm, but a few larger crystals could be found. The largest of the selected crystals (0.07× 0.01×0.01 mm) was fixed at a Mitigen MicroMesh holder with cryoil and used for a single–crystal X–ray diffraction study. Powder X–ray diffraction (Stoe Stadi MP, Cu Kα_1_, curved germanium monochromator (111), linear PSD) has proved that the selected crystal was representative for the whole powder sample batch.

### Refinement

After the positions of Bi atoms were determined by direct methods, carbon and oxygen atoms could be located from difference Fourier maps one after another in several cycles of refinement. Hydrogen atoms of the aromatic ring were placed geometrically with C—H distance 0.93Å with *U*_iso_(H) = 1.2*U*_eq_(C). The highest peak at the electron density map is located at 1.23Å distance from Bi1 atom and could be a consequence of the Fourier sum truncation.

### Crystal data

**Table:**

[Bi(C_7_H_5_O_2_)_3_]	*D*_x_ = 2.098 Mg m^−^^3^
*M**_r_* = 572.31	Mo *K*α radiation, λ = 0.71073 Å
Trigonal, *R*3	Cell parameters from 964 reflections
Hall symbol: R 3	θ = 3.6–28.0°
*a* = 19.5608 (19) Å	µ = 9.77 mm^−^^1^
*c* = 4.0967 (5) Å	*T* = 295 K
*V* = 1357.5 (2) Å^3^	Needle, colourless
*Z* = 3	0.07 × 0.01 × 0.01 mm
*F*(000) = 816	

### Data collection

**Table:**

Oxford Diffraction Gemini R Ultra diffractometer	1355 independent reflections
Radiation source: fine–focus sealed tube	1076 reflections with *I* > 2σ(*I*)
graphite	*R*_int_ = 0.087
Detector resolution: 10.3457 pixels mm^-1^	θ_max_ = 28.0°, θ_min_ = 3.6°
ω scans	*h* = −25→20
Absorption correction: multi-scan (*CrysAlis PRO*; Oxford Diffraction, 2010)	*k* = −16→25
*T*_min_ = 0.954, *T*_max_ = 1.000	*l* = −5→5
3909 measured reflections	

### Refinement

**Table:**

Refinement on *F*^2^	Secondary atom site location: difference Fourier map
Least-squares matrix: full	Hydrogen site location: inferred from neighbouring sites
*R*[*F*^2^ > 2σ(*F*^2^)] = 0.042	H-atom parameters constrained
*wR*(*F*^2^) = 0.049	*w* = 1/[σ^2^(*F*_o_^2^) + (0.*P*)^2^] where *P* = (*F*_o_^2^ + 2*F*_c_^2^)/3
*S* = 0.76	(Δ/σ)_max_ < 0.001
1355 reflections	Δρ_max_ = 1.52 e Å^−^^3^
84 parameters	Δρ_min_ = −0.68 e Å^−^^3^
0 restraints	Absolute structure: Flack (1983), 622 Friedel pairs
Primary atom site location: structure-invariant direct methods	Flack parameter: −0.034 (12)

### Special details

**Table:**

Geometry. All s.u.'s (except the s.u. in the dihedral angle between two l.s. planes) are estimated using the full covariance matrix. The cell s.u.'s are taken into account individually in the estimation of s.u.'s in distances, angles and torsion angles; correlations between s.u.'s in cell parameters are only used when they are defined by crystal symmetry. An approximate (isotropic) treatment of cell s.u.'s is used for estimating s.u.'s involving l.s. planes.
Refinement. Refinement of *F*^2^ against ALL reflections. The weighted *R*–factor *wR* and goodness of fit *S* are based on *F*^2^, conventional *R*–factors *R* are based on *F*, with *F* set to zero for negative *F*^2^. The threshold expression of *F*^2^ > σ(*F*^2^) is used only for calculating *R*–factors(gt) *etc*. and is not relevant to the choice of reflections for refinement. *R*–factors based on *F*^2^ are statistically about twice as large as those based on *F*, and *R*–factors based on ALL data will be even larger.

### Fractional atomic coordinates and isotropic or equivalent isotropic displacement parameters (Å^2^)

**Table:**

	*x*	*y*	*z*	*U*_iso_*/*U*_eq_	
Bi1	0.0000	1.0000	0.0000	0.02741 (15)	
O1	0.0761 (3)	0.9819 (3)	0.3632 (14)	0.0364 (15)	
O2	0.0144 (3)	0.8798 (3)	0.0486 (14)	0.0401 (15)	
C1	0.0647 (5)	0.9145 (5)	0.271 (2)	0.0275 (19)	
C2	0.1132 (4)	0.8836 (4)	0.4092 (19)	0.0264 (19)	
C7	0.1798 (5)	0.9308 (5)	0.585 (2)	0.036 (2)	
H7	0.1952	0.9835	0.6222	0.043*	
C6	0.2236 (5)	0.8990 (5)	0.7059 (19)	0.038 (2)	
H6	0.2682	0.9307	0.8307	0.045*	
C3	0.0925 (5)	0.8056 (5)	0.3449 (19)	0.041 (2)	
H3	0.0483	0.7739	0.2184	0.049*	
C4	0.1376 (6)	0.7756 (5)	0.469 (2)	0.048 (3)	
H4	0.1233	0.7232	0.4309	0.058*	
C5	0.2038 (6)	0.8234 (6)	0.649 (2)	0.043 (2)	
H5	0.2349	0.8038	0.7311	0.052*	

### Atomic displacement parameters (Å^2^)

**Table:**

	*U*^11^	*U*^22^	*U*^33^	*U*^12^	*U*^13^	*U*^23^
Bi1	0.02780 (19)	0.02780 (19)	0.0267 (3)	0.01390 (9)	0.000	0.000
O1	0.035 (4)	0.030 (4)	0.043 (4)	0.015 (3)	0.005 (3)	−0.001 (3)
O2	0.034 (4)	0.045 (4)	0.042 (4)	0.020 (3)	0.000 (3)	−0.013 (3)
C1	0.027 (5)	0.028 (5)	0.030 (5)	0.016 (4)	0.006 (4)	0.007 (4)
C2	0.023 (5)	0.026 (5)	0.029 (5)	0.011 (4)	0.003 (4)	0.001 (4)
C7	0.040 (6)	0.036 (5)	0.040 (6)	0.025 (5)	0.002 (4)	0.000 (4)
C6	0.035 (5)	0.040 (6)	0.040 (6)	0.020 (5)	−0.002 (4)	0.001 (5)
C3	0.055 (6)	0.046 (6)	0.035 (6)	0.035 (5)	0.002 (5)	0.005 (5)
C4	0.066 (7)	0.035 (6)	0.056 (7)	0.035 (6)	0.009 (5)	0.014 (5)
C5	0.050 (6)	0.061 (7)	0.040 (6)	0.043 (6)	0.008 (5)	0.019 (5)

### Geometric parameters (Å, °)

**Table:**

Bi1—O1	2.254 (5)	C5—C6	1.349 (10)
Bi1—O2	2.513 (5)	C6—C7	1.379 (10)
Bi1—C1	2.782 (8)	C7—C2	1.364 (10)
O1—C1	1.280 (8)	C7—H7	0.9300
O2—C1	1.260 (8)	C6—H6	0.9300
C1—C2	1.472 (10)	C3—H3	0.9300
C2—C3	1.394 (10)	C4—H4	0.9300
C3—C4	1.378 (10)	C5—H5	0.9300
C4—C5	1.372 (12)		
			
O1—Bi1—O1^i^	81.2 (2)	C4—C5—C6	119.8 (8)
O1—Bi1—O2^i^	76.50 (17)	C5—C6—C7	122.0 (8)
O1—Bi1—O2	53.78 (18)	C6—C7—C2	118.8 (8)
O1^i^—Bi1—O2	131.88 (18)	C7—C2—C3	119.8 (8)
O2^i^—Bi1—O2	119.38 (4)	C2—C7—H7	120.6
C1—O1—Bi1	100.2 (5)	C6—C7—H7	120.6
C1—O2—Bi1	88.5 (4)	C5—C6—H6	119.0
O2—C1—O1	117.2 (7)	C7—C6—H6	119.0
O2—C1—C2	123.1 (7)	C4—C3—H3	120.0
O1—C1—C2	119.5 (8)	C2—C3—H3	120.0
C1—C2—C7	121.2 (7)	C5—C4—H4	120.2
C1—C2—C3	118.9 (7)	C3—C4—H4	120.2
C2—C3—C4	120.0 (8)	C6—C5—H5	120.1
C3—C4—C5	119.6 (8)	C4—C5—H5	120.1
			
O1^i^—Bi1—O1—C1	165.2 (4)	O2—C1—C2—C7	163.1 (8)
O1^ii^—Bi1—O1—C1	82.8 (5)	O1—C1—C2—C7	−12.8 (11)
O2^i^—Bi1—O1—C1	−140.1 (5)	O2—C1—C2—C3	−13.7 (11)
O1—Bi1—O2—C1	−3.1 (4)	O1—C1—C2—C3	170.4 (7)
O2^ii^—Bi1—O1—C1	102.3 (5)	C3—C2—C7—C6	−2.5 (12)
O1^i^—Bi1—O2—C1	−27.3 (5)	C1—C2—C7—C6	−179.2 (7)
O1^ii^—Bi1—O2—C1	−92.0 (5)	C2—C7—C6—C5	1.7 (13)
O2^i^—Bi1—O2—C1	38.8 (5)	C7—C2—C3—C4	2.4 (12)
O2^ii^—Bi1—O2—C1	−125.6 (4)	C1—C2—C3—C4	179.2 (8)
Bi1—O2—C1—O1	5.0 (7)	C2—C3—C4—C5	−1.5 (13)
Bi1—O2—C1—C2	−171.0 (7)	C7—C6—C5—C4	−0.9 (13)
Bi1—O1—C1—O2	−5.7 (8)	C3—C4—C5—C6	0.8 (13)
Bi1—O1—C1—C2	170.5 (6)		

Symmetry codes: (i) −*y*+1, *x*−*y*+2, *z*; (ii) −*x*+*y*−1, −*x*+1, *z*.
